# Three new species of *Meleonoma* Meyrick from Yunnan, China (Lepidoptera, Gelechioidea, Xyloryctidae)

**DOI:** 10.3897/zookeys.904.47189

**Published:** 2020-01-16

**Authors:** Aihui Yin, Yan Zhi, Yanpeng Cai

**Affiliations:** 1 Morphological Laboratory, Guizhou University of Traditional Chinese medicine, Guiyang 550025, Guizhou, China Guizhou University of Traditional Chinese medicine Guiyang China; 2 Laboratory Animal Center, Guizhou Medical University, Guiyang, Guizhou, 550025, China Guizhou Medical University Guiyang China

**Keywords:** Key, morphology, moth, taxonomy

## Abstract

Three new species of *Meleonoma* Meyrick, 1914 (Gelechioidea, Xyloryctidae) from China, Yunnan Province, *Meleonoma plicata***sp. nov.**, *M. scalprata***sp. nov.** and *M. taeniata***sp. nov.**, are described and illustrated. A key to *Meleonoma* species known from China is provided.

## Introduction

This paper is a continuation of our taxonomic studies of the Chinese *Meleonoma* Meyrick, 1914. The last contribution of ours to this subject was a description of two new species from China with a review of its taxonomic history (Yin and Cai 2019), and yet, the examination of newly received specimens collected from Yunnan Province brought another three new species, *Meleonoma plicata* sp. nov., *M. scalprata* sp. nov. and *M. taeniata* sp. nov., which are described and illustrated here. Adding the three Japanese species recently published by Kitajima and Sakamaki (2019), the total number of *Meleonoma* species has thus increased to 43, with 19 occurring in China. A checklist of the Chinese *Meleonoma* prior to this study can be found in Yin and Cai (2019).

Little is known about their biology. The larvae of some species, such as *M. tamraensis* Park, 2016 and *M. flavilineata* Kitajima & Sakamaki, 2019, are case-bearers. The cases are usually semi-oblong or cylindrical, and built from dead broad leaves and fragments of moss and stems of Poaceae (Kitajima and Sakamaki 2019).

As summarized in Yin and Cai (2019), a well-defined taxonomic position of the genus *Meleonoma* has not been proposed yet. In the absence of recent phylogenetic research, we continue to follow Kim et al. (2016), tentatively placing *Meleonoma* in the Xyloryctidae.

## Material and methods

The examined specimens were collected from Yunnan Province in southwestern China in 2018. The descriptive terminology of the anatomical structures generally follows Wang (2006a) and Kristensen (2003). Photographs of adults were taken using a Canon EOS 6D Mark II camera plus an EF 100 mm f/2.8L MACRO IS USM lens with the help of EOS Utility 3.10.20 software. Images of genitalia were captured using a Leica DM4 B upright microscope and photomontage was performed with Leica Application Suite X imaging software. All type specimens are deposited in the Morphological Laboratory, Guizhou University of Traditional Chinese Medicine, Guiyang 550025, Guizhou, China.

## Taxonomy

### Genus Meleonoma

Taxon classificationAnimaliaLepidopteraCosmopterigidae

Meyrick, 1914

19EDD667-DE86-5D6F-AD7A-FE065782563E

Meleonoma Meyrick, 1914: 255. Type species: Cryptolechia stomota Meyrick, 1910a, by original designation.
= Acryptolechia Lvovsky, 2010: 378. Type species: Cryptolechia malacobyrsa Meyrick, 1921. Synonymised by Lvovsky (2015).

#### Diagnosis.

See Yin and Cai (2019: 80).

##### Key to *Meleonoma* species from China

**Table:** 

1	Male genitalia with an oval process arising from junction of valva and sacculus	***M. foliata* Li, 2004**
–	Male genitalia without any process arising from junction of valva and sacculus	**2**
2	Costa of valva with a sclerotized process	**3**
–	Costa of valva smooth, without any process	**4**
3	Phallus with an irregularly shaped large cornutus	***M. echinata* Li, 2004**
–	Phallus heavily wrinkled and covered with numerous tiny spines distally (Fig. 4)	***M. plicata* sp. nov.**
4	Ventral margin of valva with rounded hairy prominence or sclerotized tooth-shaped or thornlike process	**5**
–	Ventral margin of valva smooth, without any prominence or process	**10**
5	Ventral margin of valva with rounded hairy prominence near base	**6**
–	Ventral margin of valva with sclerotized tooth-shaped or thornlike process	**7**
6	Saccus broader and shorter, triangular in shape; phallus without any sclerotized spine or cornutus in male genitalia. Ductus bursae long and nonsclerotized; signum spine-shaped in female genitalia	***M. flavimaculata* (Christoph 1882)**
–	Saccus narrower and longer, linguiform in shape; phallus with a group of sclerotized sclerites near apex in male genitalia. Ductus bursae short, lateral sides sclerotized basally; signum irregularly rounded, with toothlike spines in female genitalia	***M. peditata* (Wang 2006b)**
7	Valva with large heavily sclerotized thornlike process along median part of ventral margin	***M. margisclerotica* Wang, 2016**
–	Valva with small spinelike process on ventral margin	**8**
8	Valva with small spinelike process ventroapically	***M. torophanes* (Meyrick 1935)**
–	Valva with small spinelike process at about middle of ventral margin	**9**
9	Uncus long and slender, rodlike; sacculus somewhat triangular in shape, dorsal and ventral margins straightly converging into slightly pointed apex, without any extra process	***M. pardalias* Meyrick, 1931**
–	Uncus short, lanciform; sacculus somewhat trapezoid in shape, dorsal and ventral margins each bearing a sclerotized process respectively	***M. malacobyrsa* (Meyrick 1921)**
10	Uncus sclerotized, long and slender, rodlike, apex acute	**11**
–	Uncus membranous, short, not rodlike	**18**
11	Saccus short and broad, widely rounded at apex	**12**
–	Saccus triangular or funnel-shaped, tapered or at least narrowly rounded at apex	**13**
12	Phallus with numerous extremely small spines distally	***M. meyricki* Lvovsky, 2015**
–	Phallus without cornutus or any other spine	***M. malacognatha* Li & Wang, 2002**
13	Forewing mostly blackish brown, broadly and densely scattered with orange yellow forming irregular streaks and blotches, or only sparsely diffused with yellow	**14**
–	Forewing mostly yellow, more or less mixed with brown, with or without brown fascia or patch on apical half	**15**
14	Forewing broadly and densely scattered with orange yellow forming irregular streaks and blotches. Sacculus with several short spines distally and no extra process; phallus with a curved band-shaped plate distally	***M. apicispinata* Wang, 2016**
–	Forewing only sparsely diffused with yellow. Sacculus with a long bladelike process distally; phallus with three small teeth distally	***M. projecta* Yin, 2019**
15	Valva small, oblong and pointing downward, median surface covered with long and thick setae	***M. polychaeta* Li, 2004**
–	Valva generally broad, knife-shaped and uplifted, median surface covered with thin long hairs	**16**
16	Basal half of valva densely covered with long hairs on median surface	***M. facunda* (Meyrick 1910b)**
–	Basal half of valva only sparsely covered with long hairs on median surface	**17**
17	Dorsal margin of sacculus with one process at end; phallus straight, with one rodlike sclerite extending from middle to apex (Fig. 5)	***M. scalprata* sp. nov.**
–	Sacculus with two sclerotized processes at end of dorsal and ventral margins respectively; phallus hooked in distal 1/4, without any sclerite	***M. foliiformis* Yin, 2019**
18	Uncus triangular; sacculus without any process; phallus forming an 8-shaped bandlike structure distally (Fig. 7)	***M. taeniata* sp. nov.**
–	Uncus cone-shaped; sacculus with three processes; phallus with one short cornutus in vesica	***M. facialis* Li & Wang, 2002**

### Meleonoma plicata sp. nov.

Taxon classificationAnimaliaLepidopteraCosmopterigidae

FA5154EC-F907-5A18-A884-D1231E47718D

http://zoobank.org/FEF920AC-F5FD-4DDF-936B-4DC7D43F5035

Figs 1 , 4

#### Material examined.

***Holotype***: China • ♂; Yunnan Province, Puer City, Lianhua Village; alt. 1300 m, 20 May 2018; Yan Zhi leg.; YAH19068.

#### Diagnosis.

This new species, *M. foliiformis*, *M. malacobyrsa* and *M. scalprata* share many characters in both appearance and male genitalia, which implies that these four species might belong to the same lineage within *Meleonoma*. In appearance, they all have a relatively large wingspan; similar composition of body color (primarily yellow and brown); similar pattern of coloration of forewings (mostly yellow, more or less mixed with brown near base, a brown fascia from costa obliquely to slightly before tornus, a somewhat triangular brown patch at apex). In male genitalia, they all share the following characters: valva large and broad with median surface densely covered with long hairs; sacculus broad, nearly triangular or trapezoid with various sclerotized processes. However, *M. plicata* can be easily distinguished from the others by the following character combination: forewing with basal 1/3 densely mixed with blackish brown speckles; dorsal margin of valva with a small finger-shaped process at distal 1/6, ventral margin smooth, without any process; dorsal margin of sacculus with a large fingerlike process at end; phallus with an oblique portion in distal 1/3 heavily wrinkled and covered with numerous tiny spines.

#### Description.

***Head***: vertex and front pale gray, mixed with yellow bilaterally; labial palpus long and recurved, extending well beyond vertex, with smooth scales, yellow, segment 1 mixed with dark brown on outer surface, segment 2 blackish brown distally and extending to middle of ventral margin; segment 3 about 3/4 length of segment 2; antenna with scape blackish brown on dorsal surface and yellow on ventral surface, with flagellum ringed, alternately blackish brown and yellow, except almost pure pale yellow on ventral surface of basal half flagellomeres; scales of proboscis yellow.

**Figures 1–3.**
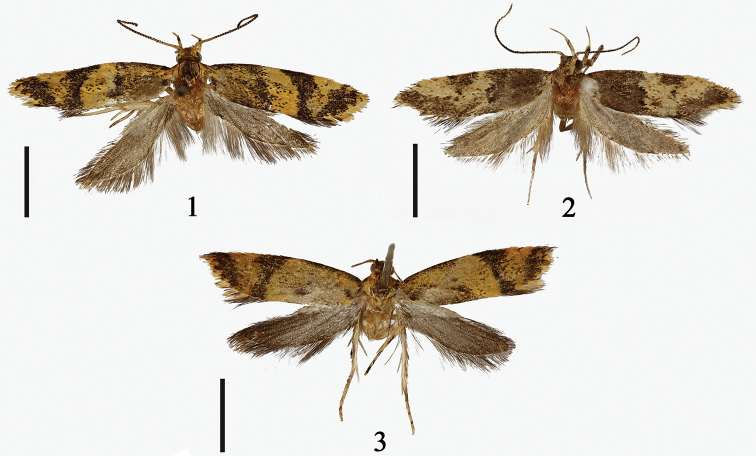
*Meleonoma* species, morphology **1** adult of *M. plicata* sp. nov., holotype, male (gen. slide no. YAH19068) **2** adult of *M. taeniata* sp. nov., holotype, male (gen. slide no. YAH19071) **3** adult of *M. scalprata* sp. nov., paratype, female (gen. slide no. YAH19070). Scale bars: 2.50 mm.

***Thorax***: tegula and mesonotum blackish brown mixed with yellow; legs whitish yellow, tibiae and tarsi scattered with blackish brown speckles on outside. Forewing (Fig. 1): length 5.8 mm (*N* = 1), about 3.6 × as long as wide, yellow, basal 1/3 quite densely mixed with blackish brown speckles; a blackish brown fascia extending from basal 3/5 of costa obliquely to slightly before tornus, with inner margin slightly arched outward, outer margin somewhat serrated irregularly; cell with two dim black dots, one set at middle, other at middle of fold; apex forming a somewhat triangular patch, blackish brown, mixed with yellow along apex and termen; other yellow parts sparsely scattered with blackish brown scales; cilia yellow except blackish brown on tornus; ventral surface yellowish brown. Hindwing (Fig. 1): translucent grayish brown, gradually darkening towards apex; cilia grayish.

**Figures 4–7.**
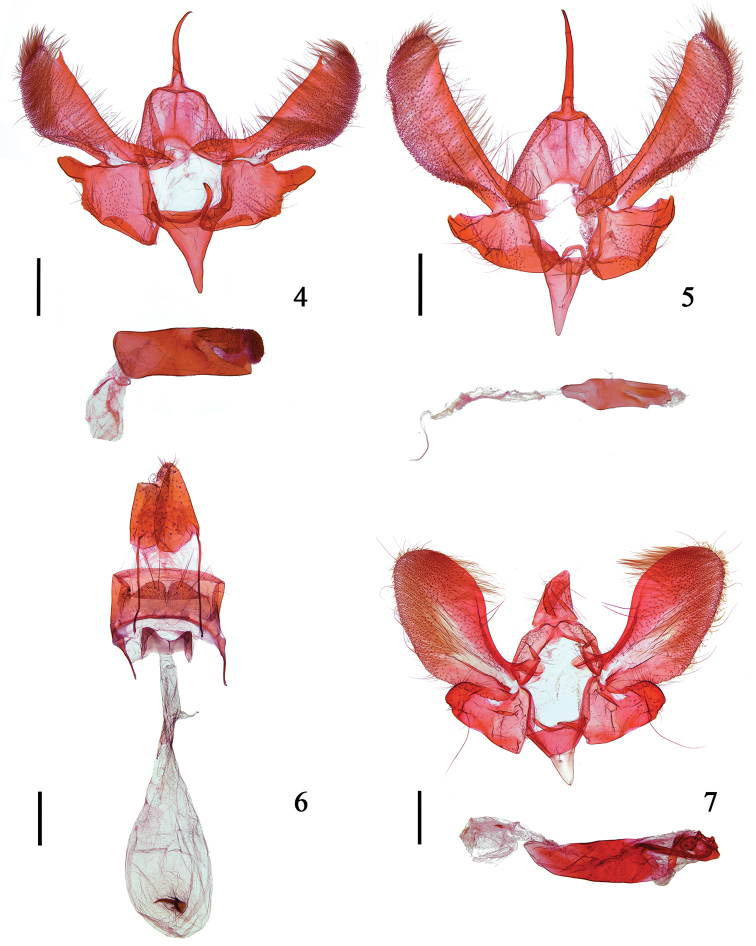
*Meleonoma* species, morphology **4** male genitalia of *M. plicata* sp. nov., holotype, phallus illustrated separately (gen. slide no. YAH19068) **5** male genitalia of *M. scalprata* sp. nov., holotype, phallus illustrated separately (gen. slide no. YAH19069) **6** female genitalia of *M. scalprata* sp. nov., paratype (gen. slide no. YAH19070) **7** male genitalia of *M. taeniata* sp. nov., holotype, phallus illustrated separately (gen. slide no. YAH19071). Scale bars: 0.25 mm.

***Male genitalia*** (Fig. 4): uncus with base short, slightly dilated bilaterally, with other part quite long and slender, slightly curved, apex acute; gnathos mostly membranous, with lateral arms arched outward; tegumen near bell-shape, lateral arms about same width, posterior margin slightly concave at middle, anterior margin shallowly concave into parenthesis-shape; valva gradually widening to middle from a narrow base, with ventral margin broadly arcuate in distal half into rounded apex, median surface densely covered with long hairs; costa nearly straight, strongly sclerotized except only weakly so in distal 1/6, with a small finger-shaped process protruded outward at distal 1/6; transtilla short and weakly sclerotized, covered with rows of long hairs, protruded forward medially; sacculus broad, nearly triangular, with basal 2/3 of dorsal margin joined with valva, a large fingerlike process at end of dorsal margin, distal half of ventral margin slightly serrated, somewhat protruded, densely covered with long hairs and as well on central area of sacculus; saccus funnel-shaped narrowly rounded at apex; juxta arcuate; phallus moderately sclerotized, cigar-shaped, with an oblique portion in distal 1/3 heavily wrinkled and covered with numerous tiny spines.

***Female genitalia***: unknown.

#### Biology.

Nothing is known about the larva. The adult was collected at night in May.

#### Distribution.

Known only from the type locality (Southwest China: Yunnan Province).

#### Etymology.

The specific name is derived from the Latin adjective *plicatus* (wrinkled, folded), referring to the heavily wrinkled distal part of the phallus in male genitalia.

### Meleonoma scalprata sp. nov.

Taxon classificationAnimaliaLepidopteraCosmopterigidae

12D733DB-B69A-50DF-BADC-91A1D0BC7E6D

http://zoobank.org/CCA1F194-3A1D-4FF6-A2C9-2936B97702AF

Figs 3 , 5 , 6

#### Material examined.

***Holotype***: China • ♂; Yunnan Province, Puer City, Lianhua Village; alt. 1300 m, 20 May 2018; Yan Zhi leg.; YAH19069. ***Paratype***: 1 ♀, same collection data as for preceding; YAH19070.

#### Diagnosis.

This new species belongs to the lineage comprising *M. foliiformis*, *M. malacobyrsa* and *M. plicata*. The new species can be easily distinguished from the others by the following combination of characters: forewing with basal half mixed with blackish brown speckles; both dorsal and ventral margins of valva smooth, without any process; dorsal margin of sacculus with an inconspicuous beak-shaped process at end; phallus with one rodlike sclerite originating from middle and extending to apex.

#### Description.

***Head***: vertex pale gray, mixed with yellow bilaterally, front pale yellowish gray; labial palpus long and recurved, extending well beyond vertex, with smooth scales, yellow, segment 1 mixed with blackish brown on outer surface, segment 2 blackish brown distally and extending vaguely to middle of ventral margin; segment 3 about half length of segment 2; antenna with scape blackish brown on dorsal surface and yellow on ventral surface, with flagellum ringed, alternately blackish brown and yellow, except almost pure yellow on ventral surface of about basal half flagellomeres; scales of proboscis pale yellow.

***Thorax***: tegula yellow, very sparsely mixed with blackish brown laterally; mesonotum yellow, mixed with blackish brown and more strongly so on posterior half; legs whitish yellow, tibiae and tarsi scattered with blackish brown speckles on outside. Forewing (Fig. 3): length 5.4–5.6 mm (*N* = 2), about 3.3 × as long as wide, yellow, basal half mixed with blackish brown speckles; a narrow blackish brown fascia extending from basal 3/5 of costa obliquely to slightly before tornus, slightly wider anteriorly; cell with two very dim black dots, one set at end, other at middle of fold; apex forming a somewhat narrow triangular patch, blackish brown, mixed with yellow along apex and termen; other yellow parts sparsely scattered with blackish brown scales; cilia yellow except grayish brown on tornus; ventral surface dark yellowish brown. Hindwing (Fig. 3): translucent grayish brown, gradually darkening towards apex; cilia grayish brown.

***Male genitalia*** (Fig. 5): uncus with base short, dilated bilaterally into inverted T-shape, with other part quite long and slender, rodlike, apex acute; gnathos mostly membranous, with lateral arms slightly curved, a bit more sclerotized in basal half than distal half; tegumen nearly inverted V-shaped, lateral arms gradually narrowed to apex, posterior margin slightly concave at middle, anterior margin relatively shallowly concave into parenthesis-shape; valva somewhat in shape of table knife, gradually widening to basal 1/4 from a narrow base, with ventral margin arcuate in distal 1/5 into rounded apex, median surface densely covered with long hairs; costa strongly sclerotized, nearly straight, scattered with long hairs; transtilla covered with rows of long hairs, protruded forward medially, distal portion rounded; sacculus broad, nearly triangular, with basal half of dorsal margin joined with valva, dorsal margin with an inconspicuous beak-shaped process at end, ventral margin slightly more sclerotized, with a very shallow arcuate emargination from about middle to distal 1/4, with long hairs covering median portion and as well as central area of sacculus; saccus funnel-shaped, narrowly rounded at apex; juxta widely U-shaped; phallus moderately sclerotized, nearly cylindrical in shape, narrower in basal 1/3, with one rodlike sclerite originating from middle and extending to apex.

***Female genitalia*** (Fig. 6): papillae anales large and broad, setose; apophyses posterioris about 3 times length of apophysis anteriores; eighth tergum entirely sclerotized; eighth sternite with granules posteriorly, posterior margin narrowly concave at middle, forming two semiovate plates with long setae; lamella antevaginalis moderately sclerotized, capital M-shaped; ductus bursae entirely membranous; ductus seminalis originating from about middle of ductus bursae; corpus bursae large, ovate, entirely membranous; signum machete-shaped, with one extra small spine on each side near base.

#### Biology.

Nothing is known about the larva. The adults were collected at night in May.

#### Distribution.

Known only from the type locality (Southwest China: Yunnan Province).

#### Etymology.

The specific name is derived from the Latin adjective *scalpratus* (knife-shaped), referring to the machete-shaped signum in female genitalia.

### Meleonoma taeniata sp. nov.

Taxon classificationAnimaliaLepidopteraCosmopterigidae

D18F86B2-2EFC-50EA-81A9-8652662E7B20

http://zoobank.org/7190435D-430A-46A3-B99B-778AEEDCB020

Figs 2 , 7

#### Material examined.

***Holotype***: China • ♂; Yunnan Province, Puer City, Lianhua Village; alt. 1300 m, 21 May 2018; Yan Zhi leg.; YAH19071.

#### Diagnosis.

This new species is similar to *M. torophanes* superficially, but it can be distinguished from the latter by having one large earthy yellow V-shaped mark on forewing; uncus triangular; ventral margin of valva smooth, without any spine; sacculus with distal 1/4 sclerotized forming a thickened plate, without any extra process; phallus forming an 8-shaped bandlike structure distally. Whereas the latter has two large light-yellow marks on forewing; uncus lanciform; valva with a short spine ventroapically; sacculus forming a narrow process distally; phallus with a hairbrush-shaped sclerite attached with short spines.

#### Description.

***Head***: vertex and front pale earthy yellow mixed with dark brown; labial palpus long and recurved, extending well beyond vertex, with smooth scales, pale earthy yellow, outer surface of segment 1 dark brown, of segment 2 dark brown distally and extending vaguely to distal 2/3, of segment 3 slightly tinged with dark brown at middle, inner surface of segment 2 dark brown distally; segment 3 slightly shorter than segment 2; antenna with scape dark brown on dorsal surface and pale earthy yellow on ventral surface, with flagellum alternately dark brown and yellow on dorsal surface, except middle 1/3 flagellomeres almost pure dark brown, ventral surface pale earthy yellow; scales of proboscis pale earthy yellow.

***Thorax***: tegula and mesonotum dark brown mixed with pale earthy yellow; legs pale earthy yellow, forelegs somewhat segmented with wide dark brown rings, mid and hindlegs with tibiae and tarsi scattered with dark brown speckles on outside. Forewing (Fig. 2): length 5.5 mm (*N* = 1), about 3.6 × as long as wide, dark brown; cell with three indistinct black dots, one set at middle, one at end and one at middle of fold; a broad somewhat V-shaped mark with two ends extending from about basal 1/2 and 4/5 of costa respectively, and converging slightly before tornus, earthy yellow in color, and sparsely tinged with yellowish brown and dark brown scales; apex and termen narrowly edged with pale earthy yellow; cilia earthy yellow mixed with pale brown; ventral surface light brown. Hindwing (Fig. 2): translucent light grayish brown, gradually darkening towards apex; cilia light grayish brown.

***Male genitalia*** (Fig. 7): uncus membranous, triangular in shape, with long setae on dorsal surface; gnathos absent; tegumen inverted U-shaped, lateral arms long, about same width, posterior margin with a shallow V-shaped notch at middle, anterior margin deeply concave; valva broad, gradually widening to basal 2/5 from a relatively narrow base, with distal 3/5 nearly same width, apex broadly rounded, densely covered with long hairs on median surface, but asetose in an elongate membranous area at center; costa moderately sclerotized, broadly arched forming a shallow notch; transtilla round, narrow at base, swollen distally, asetose, protruded forward medially; sacculus broad, trapezoid, with dorsal margin joined with valva at base, with distal 1/4 strongly sclerotized forming a distinct thickened plate that sparsely covered with long setae on both outer and median surfaces, dorsal and ventral margins nearly parallel; saccus short, funnel-shaped narrowly rounded at apex; juxta bifurcated, weakly joined at base; phallus moderately sclerotized, rodlike, narrow at base, gently thickened to basal 2/3, heavily sclerotized in distal 1/3, forming a bandlike structure similar to Arabic numeral “8” in shape.

***Female genitalia***: unknown.

#### Biology.

Nothing is known about the larva. The adult was collected at night in May.

#### Distribution.

Known only from the type locality (Southwest China: Yunnan Province).

#### Etymology.

The specific name is derived from the Latin adjective *taeniatus* (bandlike), referring to the bandlike structure of the phallus in male genitalia.

## Supplementary Material

XML Treatment for Meleonoma

XML Treatment for Meleonoma plicata

XML Treatment for Meleonoma scalprata

XML Treatment for Meleonoma taeniata
